# Hormone-Dependent Bacterial Growth, Persistence and Biofilm Formation – A Pilot Study Investigating Human Follicular Fluid Collected during IVF Cycles

**DOI:** 10.1371/journal.pone.0049965

**Published:** 2012-12-04

**Authors:** Elise S. Pelzer, John A. Allan, Christina Theodoropoulos, Tara Ross, Kenneth W. Beagley, Christine L. Knox

**Affiliations:** 1 Institute of Health and Biomedical Innovation, Faculty of Science and Technology, Queensland University of Technology, Brisbane, Queensland, Australia; 2 The Wesley Research Institute, Women’s Health Laboratory, The Wesley Hospital, Auchenflower, Queensland, Australia; 3 The Wesley Reproductive Medicine Unit, The Wesley Hospital, Auchenflower, Queensland, Australia; University of Padova, Medical School, Italy

## Abstract

Human follicular fluid, considered sterile, is aspirated as part of an *in vitro* fertilization (IVF) cycle. However, it is easily contaminated by the trans-vaginal collection route and little information exists in its potential to support the growth of microorganisms. The objectives of this study were to determine whether human follicular fluid can support bacterial growth over time, whether the steroid hormones estradiol and progesterone (present at high levels within follicular fluid) contribute to the *in vitro* growth of bacterial species, and whether species isolated from follicular fluid form biofilms. We found that bacteria in follicular fluid could persist for at least 28 weeks *in vitro* and that the steroid hormones stimulated the growth of some bacterial species, specifically *Lactobacillus* spp., *Bifidobacterium* spp. *Streptococcus* spp. and *E*. *coli*. Several species, *Lactobacillus* spp., *Propionibacterium* spp., and *Streptococcus* spp., formed biofilms when incubated in native follicular fluids *in vitro* (18/24, 75%). We conclude that bacteria aspirated along with follicular fluid during IVF cycles demonstrate a persistent pattern of growth. This discovery is important since it can offer a new avenue for investigation in infertile couples.

## Introduction

Follicular fluid, which surrounds the oocyte during *in vivo* folliculogenesis, is a hypocoagulable, semi-viscous fluid comprising proteins, inorganic compounds, carbohydrates, mucopolysaccharides, lipids, gonadotrophins, steroid hormones, immunoglobulins, cytokines, complement components and growth factors [1], [2]. Previous studies have shown that whilst human follicular fluid has antimicrobial properties, it is still capable of supporting microbial growth *in vitro* [3], [4]. Cottell *et al*. [5] first reported the presence of bacteria within follicular fluid that had been collected at the time of trans-vaginal oocyte retrieval. In addition, in our recent study, we isolated numerous microorganisms from follicular fluid. The microorganisms were present as asymptomatic colonizers or as contaminants that were introduced into the follicular fluid at the time of trans-vaginal oocyte retrieval [6]. The collected follicular fluids did not appear cloudy/turbid, despite the presence of a mixed microflora, in contrast to acute microbial infections of other body fluids such as urine. In their native environment, the majority of bacteria exist as complex surface-attached communities [7]. This observation prompted our current study to investigate follicular fluid as a medium to support the growth of microorganisms. Since the follicular fluids collected from women undergoing IVF cycles contain high levels of estradiol and progesterone as a result of the IVF stimulation process, we hypothesised that these steroid hormones may affect the growth of microorganisms. We also hypothesised that microorganisms present in human follicular fluids could persist over time and form biofilms in the ovarian follicular fluid.

## Methods

### 1.1 Participants

From September 2007 to November 2008, couples commencing fully stimulated IVF cycles at Wesley-Monash IVF in Brisbane, Australia were invited to enrol in this study. Thirty-six follicular fluid specimens were randomly selected for testing and included equal numbers of clear and blood-stained fluids.

### 1.2 Ethics Statement

Ethical approval was obtained from the review boards of Uniting Care Health, Human Research Ethics Committee and Queensland University of Technology Human Ethics Committee. All patients provided informed written consent for their follicular fluids to be used in this study.

### 1.3 Trans-vaginal Oocyte Retrieval

Follicular fluid was collected by the IVF clinicians at the time of oocyte retrieval as previously described [6]. The IVF unit used a ‘boost’ protocol for controlled ovarian hyper-stimulation. For each study participant, the follicular fluid from the largest most accessible follicle in either the left or the right ovary was aspirated first. Follicular fluid was aspirated directly into sterile test tubes in the operating theatre. The follicular fluid specimens were aseptically transferred to a sterile culture dish to determine if there was an oocyte present. Following removal of the oocyte, the IVF scientists transferred the remaining follicular fluid to a sterile 15 mL Falcon tube for storage at −80°C. There was significant variability in the volume of follicular fluid collected from each follicle (range <1 mL–∼12 mL). Therefore, only the follicular fluid samples with a volume of greater than 5 mL were able to be tested using all assays.

### 1.4 Colony Identification and 16 S Ribosomal RNA (rRNA) PCR and Sequencing

Isolation and identification of microbial species from the 36 human follicular fluid specimens were performed as previously described [6]. Briefly, calibrated 1 µL inoculating loops were used to inoculate a range of microbiological culture media, which were incubated under 5% CO_2_ or anaerobically at 37°C for the isolation and identification of microorganisms.

DNA extraction was performed on 1 mL aliquots of each follicular fluid specimen, and then the extracted DNA used as a template for 16 S rRNA PCR. The preparation of PCR products for sequencing was performed as per the Australian Genome Research Facility instructions for the preparation of purified DNA (AGRF, St. Lucia, QLD). AGRF sequenced each purified PCR product using a Big Dye 3 sequencing technology (BDT) labelling sequencing platform. The sequence, obtained in a FASTA format was entered into the Basic Local Alignment Search Tool (BLAST, NCBI) for identification of clinical isolates.

### 1.5 Hormonal Effect on Bacterial Growth in Follicular Fluid

Twelve frozen follicular fluid aliquots were thawed, and 1 µl of each cultured on a range of solid agar plates and in thioglycollate broth containing estradiol and progesterone (Sigma Aldrich, Castle Hill, NSW), at concentrations of 375 µg/L and 800 µg/L respectively (the median concentrations reported in the follicular fluid collected from hyperstimulated women undergoing trans-vaginal oocyte retrieval) [8], [9]. Hormones were added to the culture media because these would be present in the native follicular fluids and would degrade over time in culture. Hormones were used only in combination, rather than individually, as both are present at high concentrations in the follicular fluids of women undergoing IVF treatment and trans-vaginal oocyte retrieval. Follicular fluid specimens were also cultured on the same media without the addition of hormones as controls. Agar plates were incubated either aerobically or anaerobically at 37°C. After seven days of incubation at 37°C, positive thioglycollate broths were vortexed and a sterile 1 µL calibrated loop used to subculture broth onto horse blood agar (Oxoid, Adelaide, SA) for quantification and identification of the bacteria present [6].

### 1.6 Long-term Follicular Fluid Culture

From 24 follicular fluid specimens 1 mL of follicular fluid was aliquoted aseptically into 1.7 mL microcentrifuge tubes and incubated at 37°C aerobically. These 24 specimens were vortexed and subcultured daily: 1 µL of the follicular fluid was subcultured, onto horse blood agar and anaerobic blood agar plates (Oxoid) and incubated aerobically and anaerobically at 37°C. After (24 hours) incubation, the number of colonies on the horse blood agar were counted and expressed as the number of colony forming units (CFU)/mL of follicular fluid.

### 1.7 Biofilm Assay

From the same 24 follicular fluid specimens 300 µL was added to a sterile 13 mm coverslip in a well of a 24-well microtiter plate for the biofilm assay. Triplicate microtiter plates (technical replicates) were prepared and subsequently incubated in aerobic and anaerobic conditions at 37°C. Two coverslips were stained as described below, and the bacteria from the third coverslip were inoculated onto media as described in Methods 1.9. The follicular fluid specimens were incubated within the microtiter plates and left undisturbed for ten days, to mimic the average period of development of follicular fluid within a maturing ovarian follicle [10]. Upon removal, coverslips were gently rinsed with PBS to remove any unbound cells. The coverslips were subsequently placed onto microscope slides and processed using a method adopted from Allison and Sutherland [11]. Briefly, the coverslips were covered with 10 mM cetyl pyridinium chloride and air-dried before heat fixation. The biofilms then were stained for 15 minutes with a 2∶1 mixture of saturated Congo red (Sigma Aldrich) solution and 10% Tween 20 (Sigma Aldrich). Slides were then rinsed, counterstained with 10% Ziehl carbol fuchsin, rinsed again and dried at 37°C. The prepared coverslips were viewed by light and confocal microscopy.

Biofilms were viewed by light microscopy using an Olympus BX41 light microscope (Olympus, Tokyo, Japan), and images captured with a MicroPublisher 3.3 RTV camera (Adept Electronic Service, Warriewood, NSW) and QCapture Pro software (QImaging, Surrey, BC). Biofilms were examined under total magnifications of ×100 and ×400.

Images of the biofilms also were acquired by a TCS SP5 confocal laser scanning microscope (Leica Microsystems, Germany) equipped with a Leica HCX PL APO CS ×10 objective, and a Leica HCX PL APO CS × 63 oil immersion objective (NA 1.4). To visualise the Congo red and carbol fuschin signal, the excitation wavelength was set at 561 nm, and the fluorescence emission was detected between 567–668 nm. A series of z stack images were acquired through a volume of 60 µm and the images analysed using LAS AF (Leica Microsystems).

Biofilm maturity was graded based on the presence of key characteristic and structural features described previously by Simmons *et al.* [12]. Biofilms were classified as grade I–planktonic cells (isolated free floating cells, not adherent to the slide) and cells adherent to the conditioning film; grade II–microcolonies and groups of cells (most likely planktonic) attached to each other; grade III–extending/growing microcolonies (towers) with extracellular matrix fibrils, creating interconnections between the microcolonies, thus giving a cobweb appearance; and grade IV–towers with subterranean channels and amorphous extracellular material, giving a honeycomb appearance between the microcolonies.

### 1.8 Biofilm Assay – Scanning Electron Microscopy (SEM)

SEM was used to visualise biofilm production for four of the follicular fluid specimens and two ATCC controls (*Lactobacillus gasseri* and *Bacteroides fragilis*). 300 µL of each of the follicular fluid specimens or ATCC overnight broth cultures was added to a single sterile 13 mm coverslip in each well of a 24-well microtiter plate. Plates were performed in dulplicate so that one set was grown for 5 days, and the other 10 days. Glass coverslips were fixed in 3% glutaraldehyde in 0.1 M sodium cacodylate buffer, pH 7.4 for 24 hours at 4°C. After primary fixation, the coverslips were washed three times for 10 minutes in 0.1 M cacodylate buffer and post fixed for one hour in either 1% Osmium tetroxide or 1% Osmium tetroxide with 0.15% ruthenium red. Post-fixation, coverslips were twice washed in water and dehydrated in an ascending ethanol series (50, 70, 90, and 100% (twice)) before drying with 100% hexamethyldisilazane (HMDS) (Sigma Aldrich). Specimens were mounted on aluminium stubs with adhesive carbon tape and then sputter coated with 10 mm of gold (Leica SCD005 sputter coater). Examination of samples was performed using a FEI Quanta 3D Focused Ion Beam SEM, operating at 10 kv.

### 1.9 Biofilm Culture

The third coverslip (from Methods 1.7) was rinsed with sterile PBS; a sterile swab was used to remove the biofilm from the coverslip surface. This bacterial suspension was made in thioglycollate broth; a 1 µL calibrated inoculating loop was used to subculture the biofilm onto horse blood agar and anaerobic blood agar plates (Oxoid). Plates were incubated aerobically at 37°C for 24–48 hours and at 37°C for up to 7 days. Isolated bacterial species were identified as previously described [6].

## Results

### Follicular Fluid Culture and Bacterial Colony Identification

Bacteria were cultured from each of the 24 follicular fluid specimens tested. A single bacterial species was isolated from 15/24 (63%) of the specimens, of which 7/15 (47%) contained *Lactobacillus* species. *Propionibacterium* spp. (n = 5), *Peptostreptococcus* spp. (n = 2), or *Salmonella enterica* (n = 1) were isolated in the remaining specimens. Two bacterial species were isolated from 6/24 (25%) specimens, and five of these (83%) contained *Lactobacillus* spp. Only 3/24 (12%) follicular fluid specimens contained three bacterial species and *Lactobacillus* spp. was isolated from each of these specimens. *Lactobacillus* spp. and *Propionibacterium* spp. were the most prevalent isolates detected in follicular fluid, isolated from 51% and 14% specimens respectively (Table 1). Only 13/24 follicular fluid samples were of a sufficient volume for the complete analyses.

**Table 1:** Bacterial genera isolated and identified from cultures of follicular fluid.

Genus and species	Follicular fluid culture n = 24	Percentage of total n = 35 isolates^3^	Biofilm culture n = 18	Percentage of total n = 23 isolates^3^
*Lactobacillus gasseri* 1	9	27%	9	40%
*L. crispatus* 1	7	20%	7	30%
*L. jensenii* 1	2	5%	1	5%
CoNS^2^	3	9%	0	0%
*Propionibacterium* spp.	5	14%		
*Peptostreptococcus* spp.	2	5%	0	0%
*B. longum*	2	5%	1	5%
*S. agalactiae*	1	3%	1	5%
*S. anginosus*	1	3%	1	5%
*Micrococcus* spp.	1	3%	1	5%
*Salmonella enterica*	1	3%	1	5%
*Escherichia coli*	1	3%	0	0%
Total number of isolates	35		23	

1 *Lactobacillus* spp. were the most prevalent bacteria in follicular fluid 18/35 (51%);

2 CoNS coagulase negative staphylococci;

3 Some follicular fluids contained more than one bacterial species, giving the total number of isolates as greater than the number of follicular fluids tested.

### Hormonal Modulators of Bacterial Growth in Solid Media and Broths

When follicular fluid was cultured on solid agar media and incubated under appropriate atmospheric conditions, with or without hormone supplements, there were no differences in the number of isolated CFUs/mL (10^3^–>10^6^). However, differences were observed in the growth patterns of bacteria cultured in thioglycollate broth (with and without hormones) for 5/12 follicular fluid specimens tested (Table 2). *Lactobacillus* spp. were recovered from 7/12 (58%) thioglycollate broths. For one specimen (see Table 2, No. 12), *Lactobacillus crispatus* and *L. gasseri* were cultured from the hormone-supplemented thioglycollate media, but only *L. crispatus* was recovered from the hormone-free media. However, for the remaining *Lactobacillus* containing specimens, there were slight differences in the CFUs/mL of the *Lactobacillus* spp. isolated in the presence or absence of hormones. *Bifidobacterium* spp. was detected in two follicular fluid specimens, and where the supplemental hormones supported the growth of the *Bifidobacterium* spp., this bacterium did not grow in the absence of hormones. In contrast, the supplemental hormones inhibited the growth of *E*. *coli* and *S*. *agalactiae*, but growth was observed within the thioglycollate broth, without the addition of exogenous steroid hormones (see Table 2).

**Table 2:** Bacterial growth in hormone supplemented thioglycollate broth.

Specimen	Species identified	CFU/mL Hormone supplemented media^2^	CFU/mL Hormone-free media^2^
1	*Bifidobacterium* spp.	10^4^	No growth
2	*Bifidobacterium* spp.	10^4^	No growth
3	*E. coli*	No growth	<10^3^
4	*L. crispatus*	10^6^	10^4^
5	*L. crispatus, L. gasseri*	<10^3^ *	<10^3^
6	*L. crispatus*, *L. gasseri*	10^4^	*L. crispatus* only <10^3^
7	*L. gasseri*	10^6^	10^6^
8	*L. gasseri*	10^6^	10^4^
9	*L. gasseri*	10^6^	10^6^
10	*L. jensenii*	10^4^	10^4^
11	No growth^1^		
12	*S. agalactiae*	No growth	<10^3^

1 Culture negative, however positive by 16 S rRNA PCR assay,

2 Thioglycollate broths,

* <10^3^ represents growth in thioglycollate broth but not on solid agar subculture plates.

### Long-term Follicular Fluid *in vitro* Culture

All follicular fluid specimens cultured *in vitro* demonstrated increasing numbers of CFUs/mL at each subculture until eight days post-incubation (<10^3^–>10^6^), after which the number of cultivable bacteria reached a plateau and the numbers for these bacteria remained constant for the remaining 27 weeks (10^3^–10^6^). Furthermore, after 5–8 days incubation, only a single bacterial species in pure culture could be isolated from all follicular fluid specimens. With the exception of *S. enterica*, all of the bacterial species isolated after eight days were Gram-positive (*Lactobacillus* spp., *Bifidobacterium* spp. and *Staphylococcus* spp.). Viable Gram-positive bacteria could be recovered from these follicular fluids, which were incubated *in vitro* for 28 weeks.

### Biofilm Assay

The 24 follicular fluid specimens were tested to determine their ability to form biofilms on glass coverslips in wells of a 24-well plate. Biofilms formed *in vitro* after incubation at 37°C for 18/24 (75%) of follicular fluids. Of these biofilms, 14 were monomicrobial and four were polymicrobial as determined by the number of different colony types identified by traditional microbiological culture. The bacterial species *L. gasseri, L. crispatus, Bifidobacterium longum*, *S*. *agalactiae, S. anginosus* and *S. entericus*, if initially present in follicular fluid, were always recovered from the biofilm well sub-cultures after 10 days of *in vitro* culture. Other bacterial species, including CoNS, *Peptostreptococcus* spp. and *E. coli*, were only cultured from the original follicular fluid specimens. In 16/24 (66%) of the follicular fluids tested, the primary culture demonstrated polymicrobial colonisation, but only a single species was detected after 10 days of incubation within each well of the microtiter plate. In addition, if only a single species was detected in the primary follicular fluid culture, the same single species was always detected in the microtiter well culture. The facultative anaerobic species, *Staphylococcus* spp., *Streptococcus* spp. and *Lactobacillus* spp., were isolated following incubation under both aerobic and anaerobic conditions. In contrast, the strict anaerobes *Bifidobacterium* spp., and *Peptostreptococcus* spp. were isolated only when the follicular fluid specimens were incubated anaerobically.

**Figure 1:**
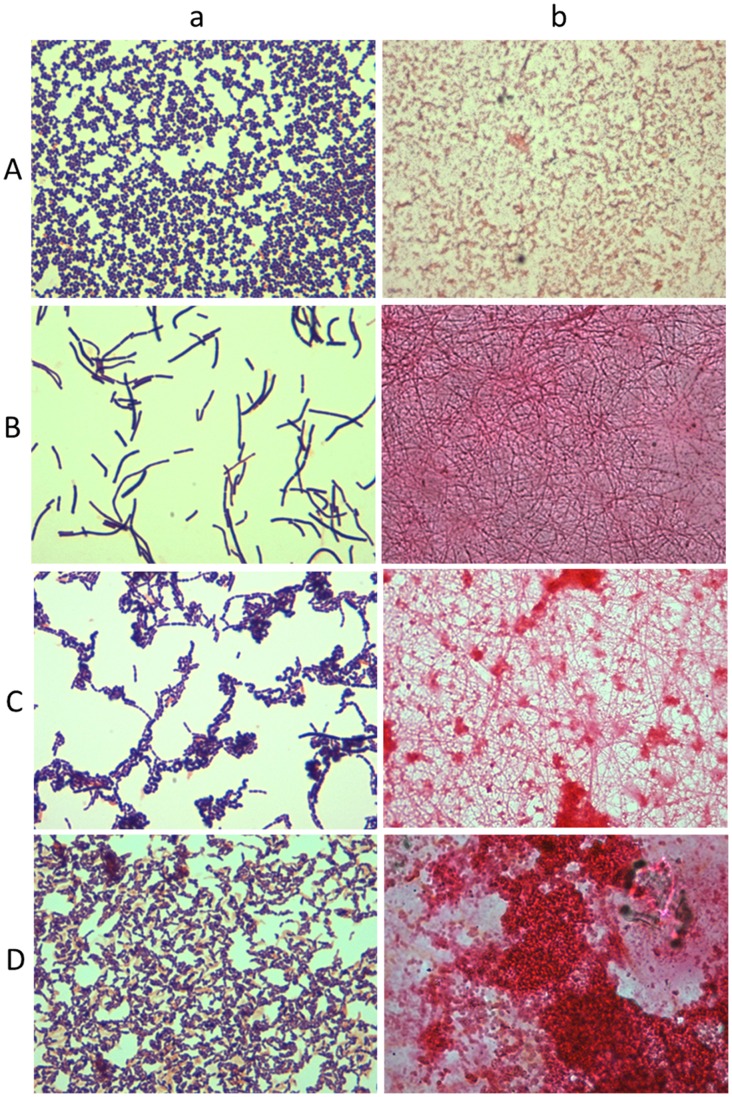
Light micrographs of Gram stains and biofilm slides. A–D (a)) Light microscopy image at ×1000 total magnification of Gram stained bacterial *S*. *agalactiae* and *Lactobacillus* spp. colonies cultured from biofilms. (A–D (b)) Light microscopy image at × 1000 total magnification of Congo red stained *S*. *agalactiae* and *Lactobacillus* spp. biofilms grown for 10 days on glass coverslips.

**Figure 2:**
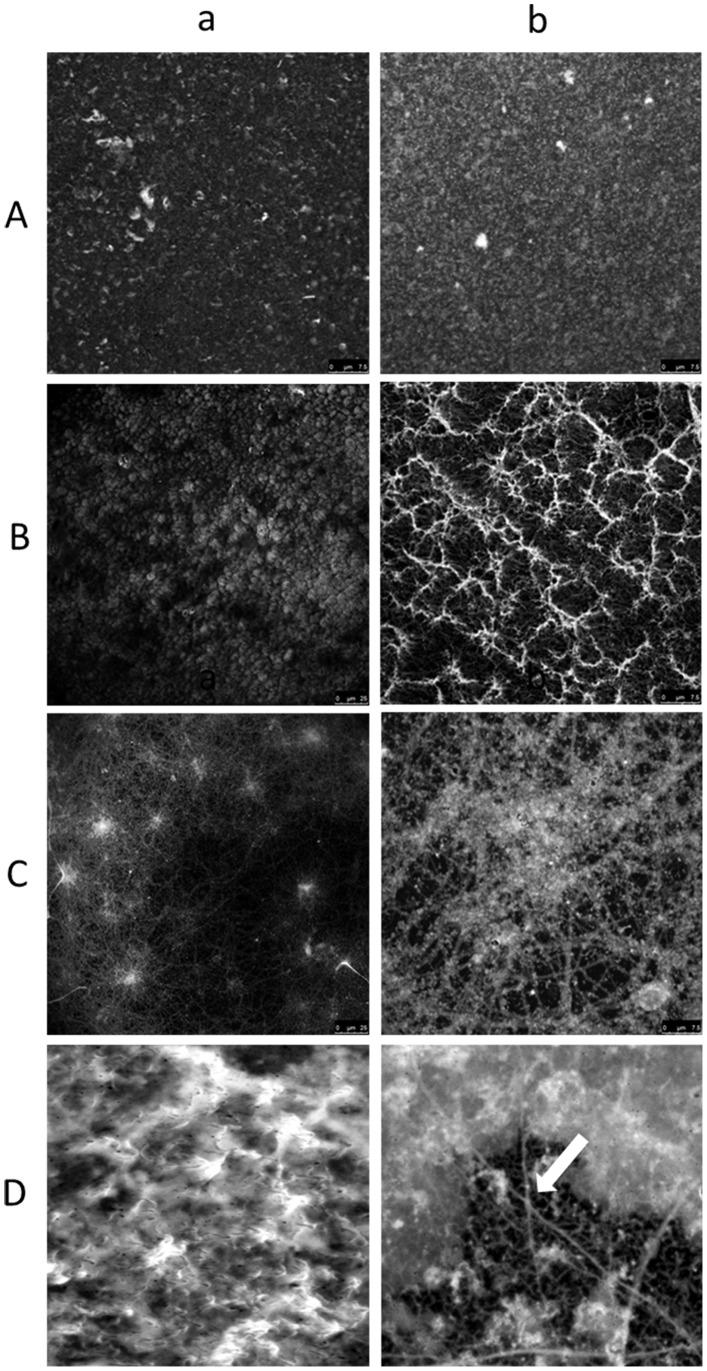
Laser scanning confocal microscopy images of biofilms. (A–D (a)) Laser scanning confocal microscopy image of various grades of biofilms at × 630 total magnification. Scale bars represent 25 µM (A–D (a) Laser scanning confocal microscopy images of various grades of biofilms at × 630 total magnifications plus × 4 zoom. Scale bars represent 7.5 µM (C (b)) Image of cobwebbing. (D (b)) Image of the honeycombed region. The arrow points to the cavities in the honeycombs. Grade II biofilms presented in Figure 2 A (a and b) demonstrated few microcolonies, some of which extended to form towers. A grade III biofilm was characterised by a carpet-pile appearance with some microcolonies extending to form towers (Figure 2B (a)). Grade IV biofilms had a honeycomb appearance where the continuous layers of cells covered the coverslip with clusters forming prominences (Figure 2D (a) and (b)).

**Figure 3:**
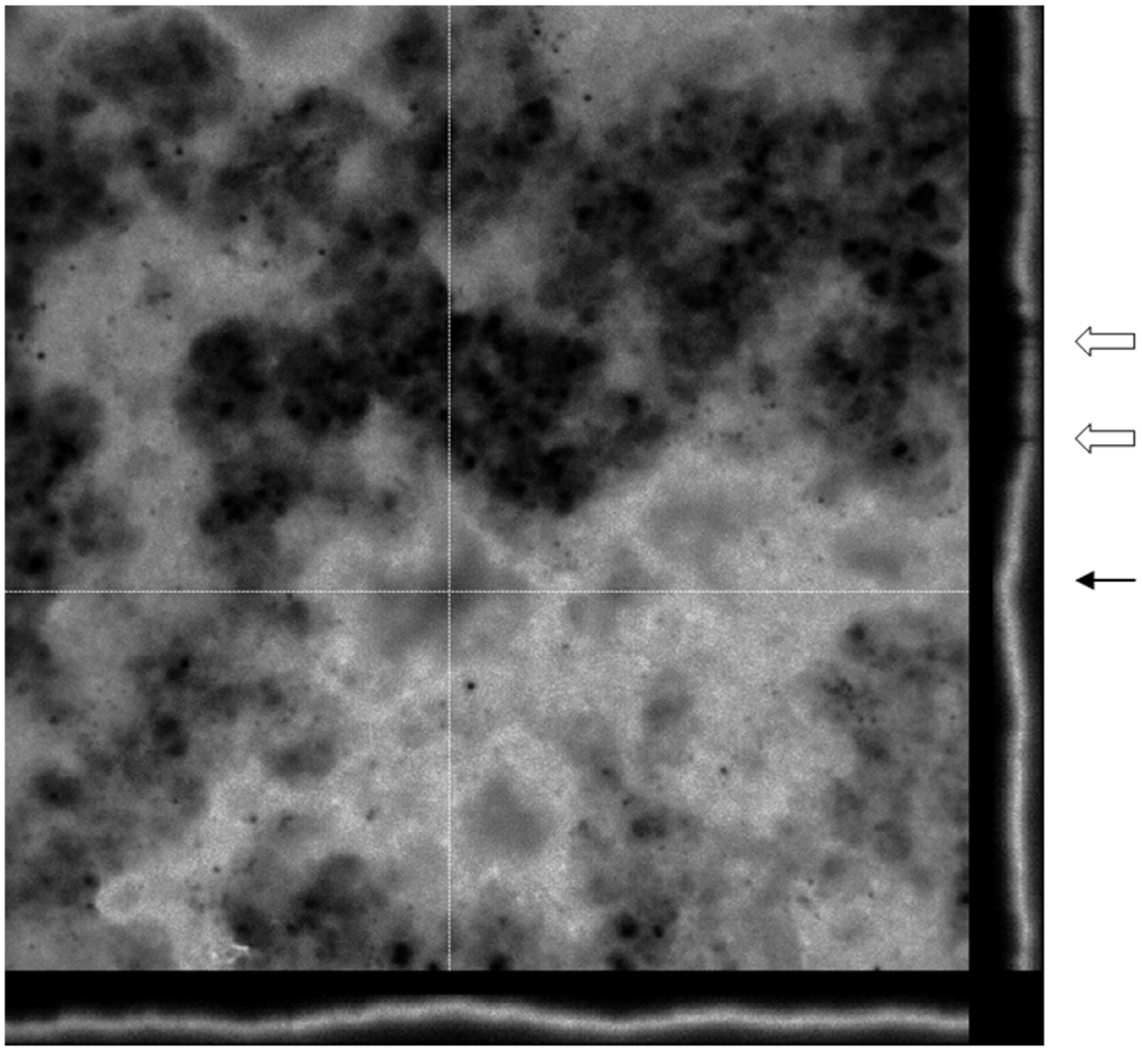
Representative orthogonal view of a 10-day-old biofilm incubated under anaerobic conditions. Laser scanning confocal microscopy image of a mature Grade IV biofilm at × 630 total magnifications plus × 4 zoom. The orthogonal view allows the representation of the 3D biofilm to be presented in 2D. The cross hairs indicate the area of the biofilm presented in 2D along the borders of the image. Depicted below and to the right of the main image are the yz and xz planes respectively. In this biofilm, there are channels between the microcolonies appearing in ‘gaps’ in the 2D structure and indicated by the white arrows. The hollow interconnecting areas under the towers are indicated by the black arrow.

For six biofilms slides, there was no evidence of biofilm formation or planktonic cell adhesion. Instead, the Congo red stain revealed the presence of a polysaccharide-containing conditioning film. The images shown in Figure 1 are representative of the different types of *Lactobacillus* spp. biofilms observed for follicular fluids incubated both under aerobic and anaerobic atmospheric conditions. Variations in the architecture of mature biofilms was observed, ranging from flat homogenous cell layers through to cell clusters, microcolonies and towers with channelling and significant quantities of amorphous extracellular material between the more complex heterogeneous structures (Figure 1 B–D). The simplest biofilm architecture visualised by light microscopy was seen for follicular fluid cultures that were incubated aerobically. The grade II biofilm presented in Figure 1A (b) demonstrated few microcolonies (which proliferate at fixed positions [13], [14], and some of these extended to form towers (outward growing masses of bacterial cells). Initial microcolonies were formed by cells growing outwards into towers, with the highest points of elevation appearing white in greyscale using confocal microscopy, as shown in Figure 2A (a) and (b). A grade III biofilm was characterised by fibril formation, as observed by light microscopy, following anaerobic incubation (Figure 1B (b)); with a carpet-pile appearance as observed with confocal microscopy with some microcolonies extending to form towers, as shown in Figure 2B (a). The polymeric extracellular matrix appeared to be more abundant in microcolonies (Figure 1C (b)). When magnified (×4 zoom, confocal microscopy), crater-like formations were visible (Figure 2B (b)). In the grade IV biofilm grown under anaerobic conditions, complex networks were observed between the microcolonies and towers and these had a cobwebbed appearance, as shown in Figure 2C (a) and (b). Grade IV biofilms were also visualised using light microscopy (Figure 1D (b)) and an amorphous polymeric extracellular matrix was observed surrounding the interconnecting microcolonies, towers and cells. In these biofilms, continuous layers of cells covered the coverslip, with clusters forming prominences. These biofilms had a honeycomb appearance, as shown in Figure 2D (a) and (b) and could be visualised using three dimensional and orthogonal confocal images. This image (Figure 3) demonstrated the presence of channels within the biofilms and hollow areas under the towers. All observed biofilms showed an uneven spatial distribution, which is consistent with the previously described characteristic of *in vitro* biofilms [15].

### Biofilm Assay – Scanning Electron Microscopy

**Figure 4:**
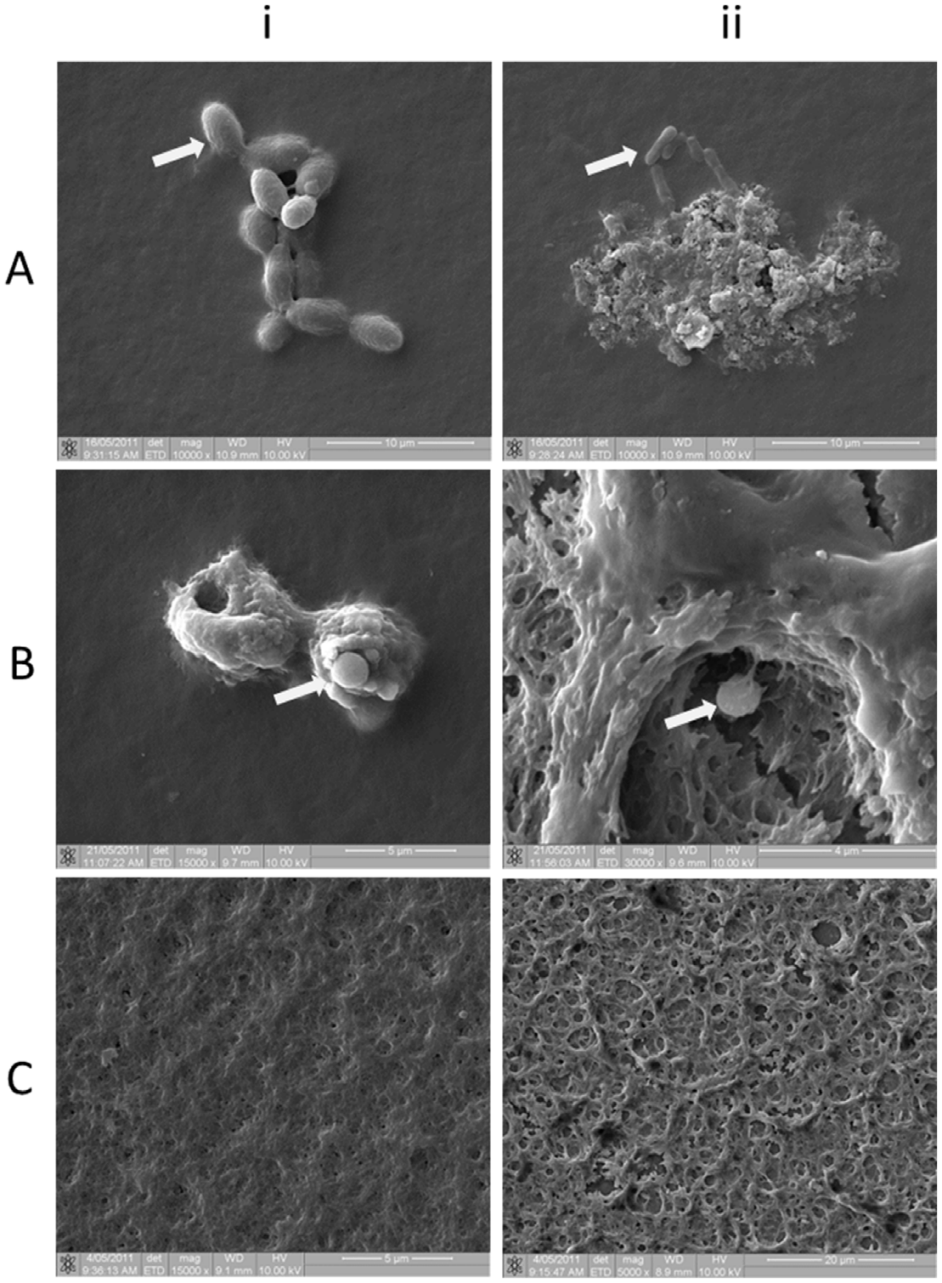
Scanning electron micrographs of 10-day-old biofilms. Individual bacilli, identified as *Lactobacillus* spp. (day 5) (arrows) are covered by a light glycocalyx (4A (i)) and a control ATCC strain *Bacteroides* spp. (day 5) by a more mature biofilm (4A (ii)) both captured at × 10000 magnification. Figures 4B (i (day 5) and ii (day 10)) represent osmium fixed biofilms containing coccoid bacteria identified as *Streptococcus* spp. (by culture) (arrows) at × 15000 and × 30000 magnification. Glycocalyx is not well preserved using traditional aldehyde fixatives followed by OsO_4_ post-fixation. Figure 4C (i) (day 10) was fixed using OsO_4_, which stabilises lipids and figure 4C (ii) (day 10) was fixed using an aldehyde fixative followed by OsO_4_ plus ruthenium red to enhance the preservation of the anionic polysaccharides in the glycocalyx.

Scanning electron microscopy (SEM) was used to visualize the surface topography of biofilms grown *in vitro* on glass coverslips for 5 days or 10 days. SEM revealed the presence of bacterial cell aggregates covered by a thin layer of glycocalyx (Figure 4A (i)), as well as microcolonies (Figure 4A (ii)). The surface topography, with a crater-like appearance was similar to that seen in images obtained using confocal microscopy (Figure 2B). The preservation of the glycocalyx was enhanced when the post-fixative OsO_4_ (Figure 4C (i)) was combined with ruthenium red (Figure 4C (ii)). The use of the ruthenium red enhanced the stabilization and visualization of the highly charged anionic mucopolysaccharides forming the extracellular glycocalyx. Evidence of biofilm maturity, extensive extracellular polysaccharide and microcolonies was apparent in both five and ten day-old biofilms.

## Discussion

Follicular fluids collected at the time of trans-vaginal oocyte retrieval were found to harbour bacteria and continued to support the bacterial growth *in vitro*. These results demonstrate that bacterial species that colonise follicular fluid, or gain access to the follicular fluid at the time of oocyte retrieval, may be a relevant focus of infertility investigations in couples with idiopathic infertility or repeated adverse IVF treatment outcomes.

Our study demonstrated that *in vitro*, elevated levels of estradiol and progesterone in thioglycollate broth media enhanced the growth of the high numbers of CFUs of *Lactobacillus* spp., which were originally present within follicular fluid specimens. This is consistent with *Lactobacillus* spp. growth in the lower genital tract. At puberty, as estrogen levels increase, the vaginal pH drops and *Lactobacillus* spp. dominate the vaginal microflora [16]. After menopause, the vaginal microflora reverts to the pre-menarchal state and the *Lactobacillus* spp. decline [17]. However, if post-menopausal women receive estrogen replacement therapy, the vaginal pH decreases and the concentration of *Lactobacillus* spp. increases [18]. The effect of endogenous steroid hormones on lactobacilli has previously been monitored in IVF patients. Jakobsson and Forsum [19] reported that during IVF treatment, with increasing estrogen levels, three major vaginal lactobacilli (*L. crispatus*, *L. gasseri* or *L. jensenii*) were predominant. These results are consistent with the findings reported in this paper, as these species were the only lactobacilli isolated from follicular fluid (collected prior to ovulation when estradiol levels were highest), although *Bifidobacterium* spp. was also isolated from the hormone-supplemented media. Both genera can metabolise carbohydrates from glycogen degradation in response to elevated estradiol levels, making the female genital tract a niche environment, enhancing the ability of the lactobacilli to persist and protect the genital tract epithelium from opportunistic infection [20]. *Bifidobacterium* spp., whilst traditionally accepted as members of the normal regional flora of the gastrointestinal tract, have more recently been detected in the vaginal flora of healthy women [20], [21], [22].

During an IVF treatment cycle, hormones including follicle stimulating hormone and human chorionic gonadotropin are administered to trigger the simultaneous maturation of multiple ovarian follicles. This results in the production of elevated levels of estradiol and progesterone within the follicular fluids collected from women undergoing trans-vaginal oocyte retrieval for IVF at levels approximately eight and three times higher, respectively, than those of women with normal cycles [8], [9]. The ovarian granulosa cells are the dominant source of these steroid hormones. Granulosa cells may be aspirated with the follicular fluid and the oocyte at the time of trans-vaginal oocyte retrieval, however once removed from the ovary, the granulosa cells are no longer under pituitary control and so rapidly proceed to undergo apoptosis [23], [24], and do not continue to produce the steroid hormones *in vitro* as they do *in vivo* [25]. Thus, over time in culture without the addition of exogenous hormones, the impact of steroid hormone levels on microbial growth would not be observed.

Studies have demonstrated that the hormone concentration within maturing follicles is significantly higher than that found in the systemic circulation, as it is governed by the hyaluronan composed cumulus-oocyte complex [2]. In the current study, hyaluronidase-producing species (*Propionibacterium* spp., *Streptococcus* spp. and *E. coli*) were isolated from follicular fluid. The hyaluronidase virulence factor of these species could lead to poor quality cumulus cells via enzymatic hyaluronan degradation, decreased steroid hormone synthesis and ultimately to detrimental effects of these more pathogenic bacteria within the follicle. Our findings indicate that high concentrations of steroid hormones within ovarian follicular fluid can influence bacterial growth in a species-dependent manner.

We demonstrated that follicular fluid supported the survival of viable bacteria for a period of at least 28 weeks. This growth occurred without the addition of supplemental nutrients or the removal of metabolic waste products; such processes that would occur *in vivo* and may facilitate continual microbial colonisation of the follicular fluid. Gurgan *et al*. [4] found that filter-sterilised and centrifuged follicular fluid was inhibitory to the Gram-positive bacteria *S. aureus, S. agalactiae* and *L. monocytogenes*, as these species did not survive *in vitro* for longer than four days. However, Gurgan *et al*. [4] did demonstrate that follicular fluid supported the growth of a range of Gram-negative species and the yeast *Candida albicans* for up to 15 days post-inoculation. These results are inconsistent with those reported here, where predominantly Gram-positive species survived, most likely as a result of the composition of the follicular fluid, which may have been significantly altered by the filtration and centrifugation prior to inoculation. To the best of our knowledge, our study is the first continuous follicular fluid culture experiment lasting 28 weeks, indicating that follicular fluid is an excellent growth medium for microorganisms.

Many of the bacteria that we detected within follicular fluid have previously been shown to form biofilms *in vitro* when cultured on vaginal epithelial cells [26], *in vivo* on the surface of intrauterine devices [27] and in amniotic fluid sludge aspirated from women diagnosed with intrauterine infection [28]. However, despite the range and load of bacteria isolated from follicular fluid, this fluid did not have a turbid appearance, which may suggest that bacteria within the follicle were present predominantly in biofilms, rather than as planktonic bacteria. Thus, the process of trans-vaginal oocyte retrieval may result in puncture and collection of the follicle wall biofilm within the lumen of the aspiration needle, and the subsequent transfer of biofilm components, including bacteria into the follicular fluid at the time of egg retrieval.

The majority of mature biofilms in the present study revealed only a single cultivable species after 10 days, even when two different species of lactobacilli were initially isolated from the follicular fluid prior to *in vitro* incubation. This may be due to competition amongst the lactobacilli themselves, or because of alterations in adhesion. It has been reported that some bacterial species can co-colonise the surface of urogenital tract cells already colonised by lactobacilli; however, after lactobacilli are established, they can cause other species of bacterial cells to detach from the epithelium, which may offer a defence mechanism by preventing attachment of pathogens to the epithelium [29], [30]. In polymicrobial biofilms, early colonising species often promote the establishment of other species [31], [32]; however, whilst the initial interactions may be synergistic, once the biofilm is established, competition between species can result in the dominance of a single species [33]. Quorum sensing, the process by which microorganisms communicate within a population, establishes the overall population size of each representative microbial species and initiates alterations in gene expression. In addition to quorum sensing, microbial growth within the biofilm is also modulated by these metabolic cues [34]. Species production of lactic acid, succinic acid and isobutyric acid has been reported to enhance both the synergistic and competitive interactions between different microbial species within a biofilm. Acid production resulting in a pH shift, often causes a reduction in the microbial diversity, as many species are acid intolerant [35]. We hypothesise that the predominance of monomicrobial *in vitro* biofilms reported in this study might therefore be due to the lactic acid and H_2_O_2_ production by *Lactobacillus* spp.

Studies of biofilms have reported that bacteria can exist in a viable, but non-cultivable state [36]. It is therefore possible that cultures of 10-day biofilms detected only those species that were actively replicating, whereas molecular techniques targeting 16 S rRNA would be able to detect and quantify all species (both viable and non-viable) present within the biofilm. This study did report that one culture-negative follicular fluid sample did not produce any visible growth on the range of solid agar or in the thioglycollate broth; however sequencing of the 16 S rRNA PCR product identified a sequence matching an uncultured bacterial clone, again highlighting the need for molecular based techniques. To gain further understanding of an individual species contribution to a biofilm, quantitative 16 S rRNA PCR assays could be used to test cultures at various time points, in order to establish growth curves for each individual species and compare culture results to PCR assay results to identify early and late species contributing to the biofilm.

An ovarian follicle has all the characteristics necessary to support the development of a microbial biofilm. Bacterial cells that would contribute to the formation of the biofilm have been detected in this work (see Table 1 and Figure 1). On some biofilm slides there is evidence of polysaccharide accumulation (in the absence of bacterial cells), which shows that there are components of follicular fluid (substrate) that could form a conditioning film to allow microbial attachment to the inside wall of the follicle (substratum). Furthermore, the *in vitro* development of various grades of biofilms in human follicular fluid (Figure 1) is demonstrated. Mature biofilms are characterised by the development of towers and fluid–filled channels [37], [38]. Mature biofilms reportedly exhibit a variety of phenotypes due to the changes in their three-dimensional structure, occurring in response to changes in cell density, osmolarity, temperature, pH and nutrient supply [36]. We showed that even follicular fluids containing the same bacterial species demonstrated different grades of biofilms and different morphology.

On six biofilm slides, no bacteria were observed, only a conditioning film was present. This could be due to the removal of any bacterial cells (by the PBS rinse prior to staining) present in the early stage of biofilm formation prior to permanent attachment to the conditioning film. Alternatively, the follicle may have been colonised by non-biofilm forming bacterial species. Further studies examining the effect of these biofilm bacterial species on each other would therefore, be beneficial to our understanding of the impact of single species and polymicrobial biofilms on reproductive health outcomes.

The findings reported in this paper have shown that (1) the ovarian steroid hormones (estradiol and progesterone) are capable of modulating the *in vitro* growth of some microbial species; (2) follicular fluid supports the long-term survival of microorganisms; and (3) bacteria isolated from follicular fluid can form biofilms *in vitro*. Further characterisation of the microorganisms detected in follicular fluid and their metabolites will increase our understanding of the effects of follicular fluid microorganisms on oocyte quality, on IVF fertilisation and pregnancy rates, and on early pregnancy events. Knowledge of the microorganisms present within this anatomical niche may lead to an improved understanding of ovarian resistance to infection and also on IVF outcomes.
